# A Rare Hepatoblastoma in a 10‐Year‐Old Boy Presenting With Tumor Rupture and Normal Alpha‐Fetoprotein Levels: A Case Report

**DOI:** 10.1002/ccr3.73820

**Published:** 2026-10-28

**Authors:** Christine Hyasint Paul, Andrew Swallow, Vulstan James Shedura, Albert Lazaro, Daniel Kitua, Ayubu Philipo, Daudi Katwana, Andrew K. Jackson, Georvin Haule, Cameron Gaskill, Kazungu Boniface Kahindi, Musa balowa, Godbless Massawe, Ally Mwanga

**Affiliations:** ^1^ Department of Surgery Muhimbili University of Health and Allied Sciences Dar es Salaam Tanzania; ^2^ Department of Surgery Mirembe National Mental Health Hospital Dodoma Tanzania; ^3^ Department of Clinical Research, Training, and Consultancy, Ministry of Health Southern Zone Referral Hospital Mtwara Tanzania

**Keywords:** alpha‐fetoprotein, hepatoblastoma, liver resection, pediatric liver tumor, tumor rupture

## Abstract

Hepatoblastoma can rarely occur in older children and may present with tumor rupture and normal alpha‐fetoprotein levels, complicating diagnosis. Early multidisciplinary evaluation and prompt surgical management are critical to improve outcomes in atypical pediatric liver tumors.

## Graphical abstract

A 10‐year‐old boy presented with a large hepatic mass (12.0 × 11.6 × 10.6 cm), classified as PRETEXT III, despite normal alpha‐fetoprotein levels (1.28 ng/mL). Tumor rupture necessitated emergency right trisectionectomy and radical colectomy. Histopathology confirmed mixed epithelial‐mesenchymal teratoid hepatoblastoma. Unfortunately, postoperative disseminated intravascular coagulopathy resulted in death. Key takeaway: Normal alpha‐fetoprotein does not exclude hepatoblastoma in older children. Early multidisciplinary evaluation, accurate diagnosis, and timely surgical intervention are essential for managing atypical presentations and preventing life‐threatening complications.

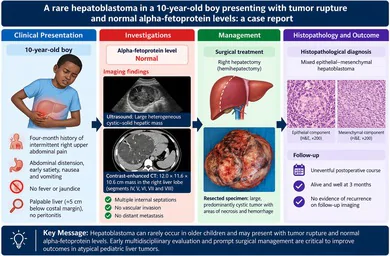

## 1 Introduction

Hepatoblastoma is a malignant tumor of embryonic origin that arises from precursor hepatocyte cells and may occasionally include epithelial, mesenchymal, and teratoid components [1, 2, 3]. It is the most common liver tumor in children under 5 years of age, with an incidence of 1.5 cases per million [4, 5]. It occurs sporadically; however, there are suggestions of increased chances for hepatoblastoma in children who were born preterm with low birth weight, in utero exposure to tobacco, familial adenomatous polyposis coli, and Beckwith–Wiedemann syndrome [6, 7, 8]. The disease has a male predilection, and the tumor commonly involves the right liver lobe [9, 10]. In this article, we describe a rare occurrence of hepatoblastoma at the age of 10 years and discuss pertinent clinical considerations.

The International Pediatric Liver Cancer Pathological Classification of 2011 subclasses hepatoblastoma as the epithelial type and the mixed epithelial and mesenchymal type. These are further subdivided into: The four epithelial subtypes are fetal, embryonal, small cell, and macrotrabecular. Where the mixed epithelial and mesenchymal subtypes are teratoid and non‐teratoid. These divergent differentiations are explained by the pluripotent stem precursor cells with the ability to differentiate [2, 11, 12].

## 2 Case History/Examination

A 10‐year‐old Black Tanzanian boy presented with a four‐month history of severe, intermittent right upper quadrant abdominal pain associated with abdominal distension, early satiety, nausea, and vomiting. However, he had normal bowel habits, no history of fevers, and no features of obstructive jaundice. The vital signs were within normal limits. Examination revealed tenderness in the right upper quadrant and a palpable liver about 5 cm below the right subcostal margin and the midclavicular line. However, no features of peritonitis were present.

Abdominal ultrasonography demonstrated hepatomegaly (liver span 16.3 cm) with a well‐circumscribed heterogeneous complex cystic mass containing both cystic and solid components within the right hepatic lobe, measuring 11.4 × 10.7 cm. The gallbladder and intrahepatic biliary ducts were unremarkable. Triphasic contrast‐enhanced CT of the chest and abdomen demonstrated an enlarged liver measuring 17.5 cm with a large predominantly hypodense heterogeneous hepatic mass measuring 12.0 × 11.6 × 10.6 cm, involving Couinaud segments IV, V, VI, VII, and VIII. The lesion contained multiple enhancing internal septations and a small peripheral enhancing solid component without internal calcifications or contrast washout. There was no evidence of portal vein thrombosis, hepatic vein or inferior vena cava invasion, biliary duct dilatation, ascites, or distant pulmonary metastases. Based on the preoperative imaging findings, the tumor was classified as PRETEXT Stage III, and multidisciplinary evaluation considered it technically resectable (Figure 1).

**FIGURE 1:**
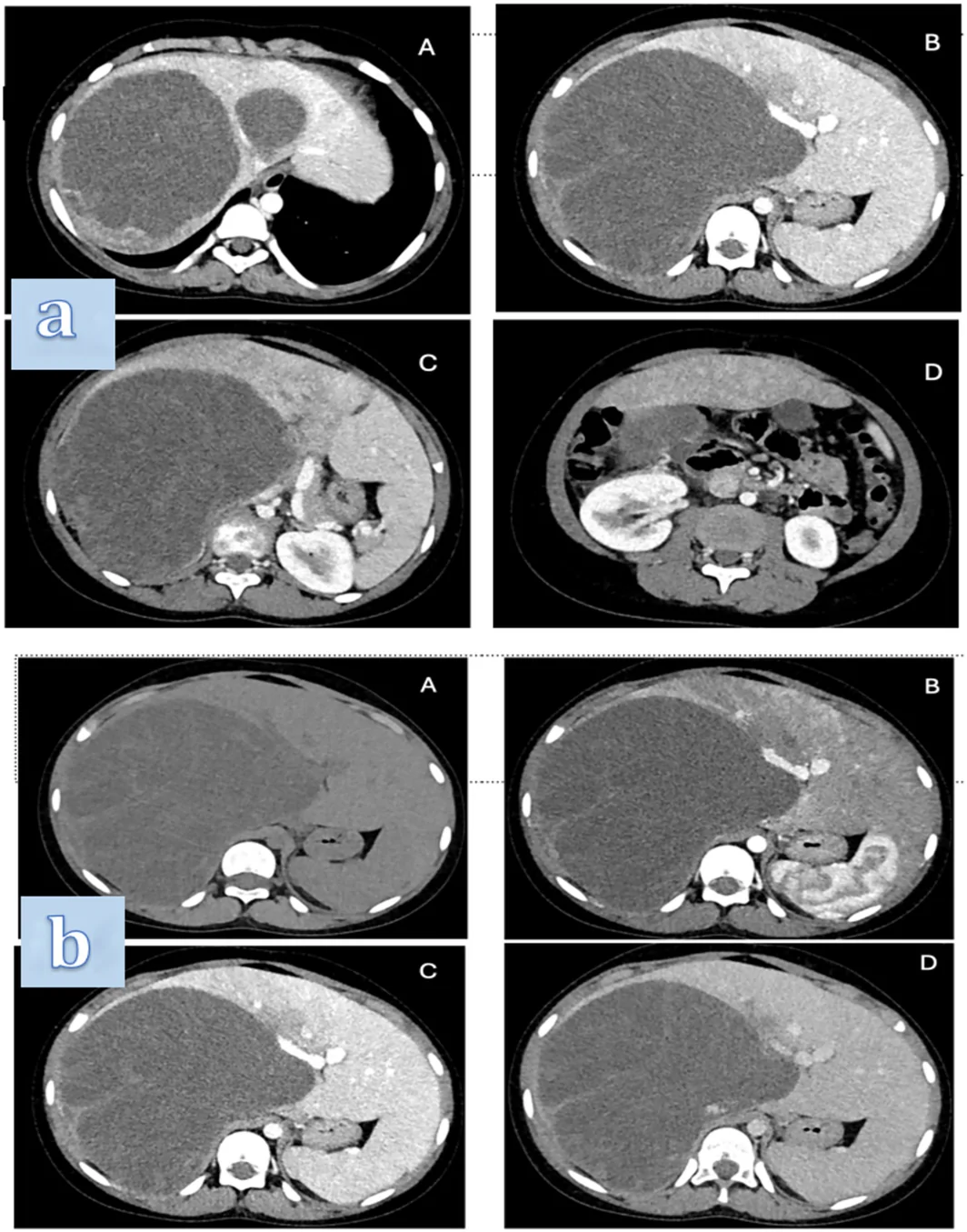
Triphasic contrast‐enhanced CT scan of the liver. (a) Portal venous phase: Axial CT images (A–C) demonstrate a large, predominantly cystic heterogeneous hepatic mass involving Couinaud Segments IV, V, VI, VII, and VIII, with multiple enhancing internal septations and a small peripheral enhancing solid component. The lesion was classified as PRETEXT stage III, indicating involvement of three contiguous liver sections with preservation of the left lateral section. There is no evidence of portal vein thrombosis, hepatic vein or inferior vena cava invasion, biliary duct dilatation, ascites, or imaging features of cirrhosis. No pulmonary metastases are identified on the chest CT images. Image D demonstrates an extrahepatic arterial supply to the tumor arising from branches of the superior mesenteric artery. (b) Triphasic CT evaluation: Axial images obtained during the A, non‐contrast (plain); B, late arterial; C, portal venous; and D, delayed phases demonstrate a thinly encapsulated, predominantly hypodense heterogeneous hepatic mass with enhancing internal septations and a peripheral solid component. No internal calcifications or contrast washout is observed. The imaging findings, together with the absence of major vascular invasion or distant metastases, were consistent with a resectable PRETEXT III hepatoblastoma, although hydatid cyst remained an important differential diagnosis before histopathological confirmation.

His full blood count was significant for hemoglobin at 8.9 g/dL. Hypoalbuminemia was present (24 g/L); however, the rest of the liver functions were preserved. He also had normal liver functions and tested negative for hepatitis B, C, and HIV. Alpha‐fetoprotein levels were also within the normal limits (1.28 ng/mL). Moreover, immunological studies for hydatid cysts tested negative.

Following multidisciplinary team discussion, the patient was planned for surgical resection based on a radiologically resectable PRETEXT III tumor. However, clinical deterioration during preoperative optimization secondary to tumor rupture necessitated emergency hepatic resection. Intraoperative findings included a large ruptured hemorrhagic cystic mass with extensive necrosis involving almost the entire right liver lobe. The mass had locally infiltrated the colonic hepatic flexure and Gerota's fascia. Hence, a right trisectionectomy and radical colectomy were performed en bloc (Figure 2). The patient was managed postoperatively in the immediate intensive care unit. However, his postoperative course was complicated by disseminated intravascular coagulopathy, from which he succumbed.

**FIGURE 2:**
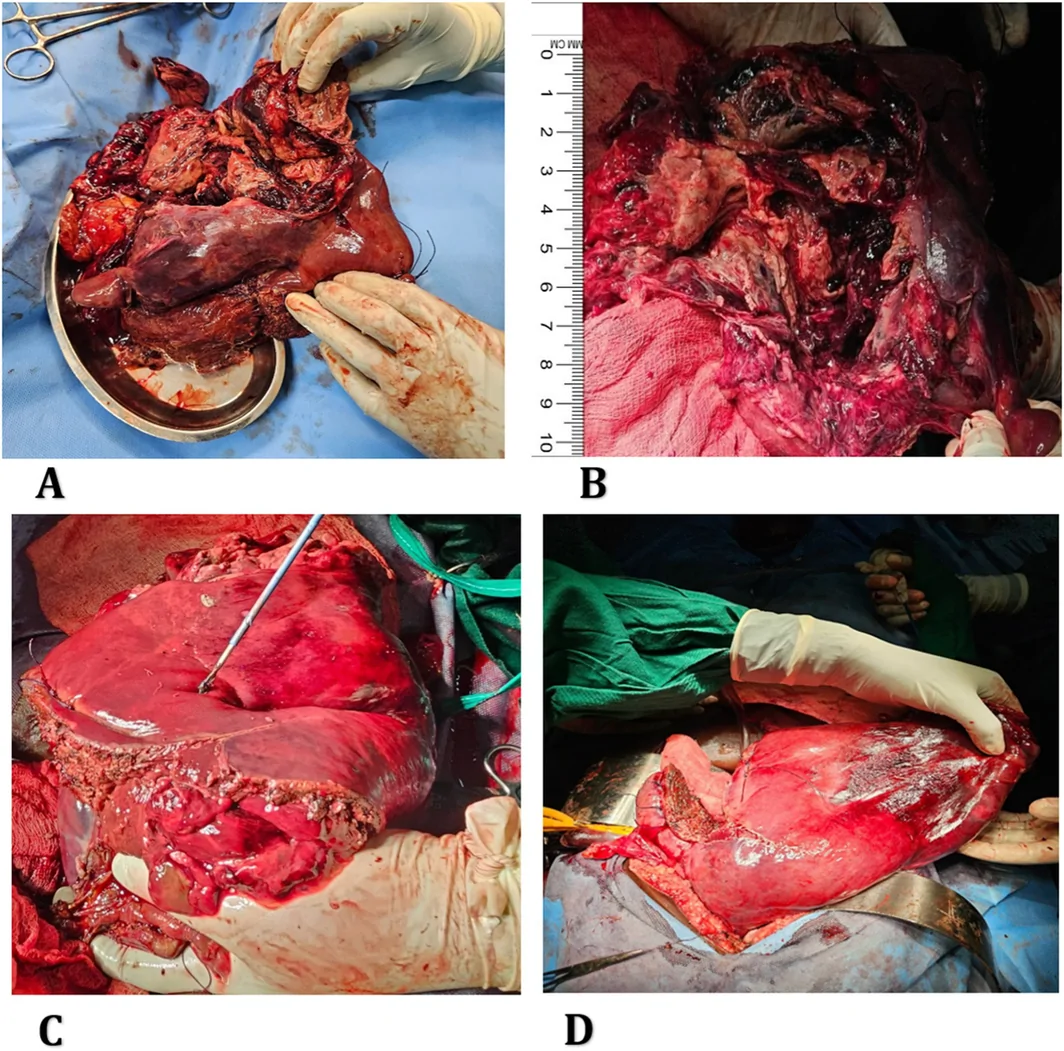
Gross photograph of the right trisectionectomy specimen. Images A and B demonstrate a large, predominantly cystic tumor with extensive areas of necrosis and hemorrhage. Images C and D show the lesion extending into the adjacent abnormal‐appearing hepatic parenchyma. The resected specimen measures approximately 19 × 10 × 10.5 cm.

Histopathological examination revealed extensive necrosis and hemorrhage. The tumorous component comprised mixed oval to spindle cells with minimal cytoplasm and vesicular chromatin. Sinusoidal structures with a trabecular arrangement of tumor cells were also observed (Figure 3). There was evidence of vascular invasion, and the resection margins were positive. Immunohistochemical analysis demonstrated diffuse positivity for Glypican‐3 and β‐catenin, focal positivity for HepPar‐1, cytokeratin AE1/AE3 expression in the epithelial component, vimentin positivity in the mesenchymal component, and negative AFP immunostaining, supporting the diagnosis (Figure 4). A final diagnosis of mixed epithelial–mesenchymal teratoid hepatoblastoma was established (Figure 3 and Figure 4).

**FIGURE 3:**
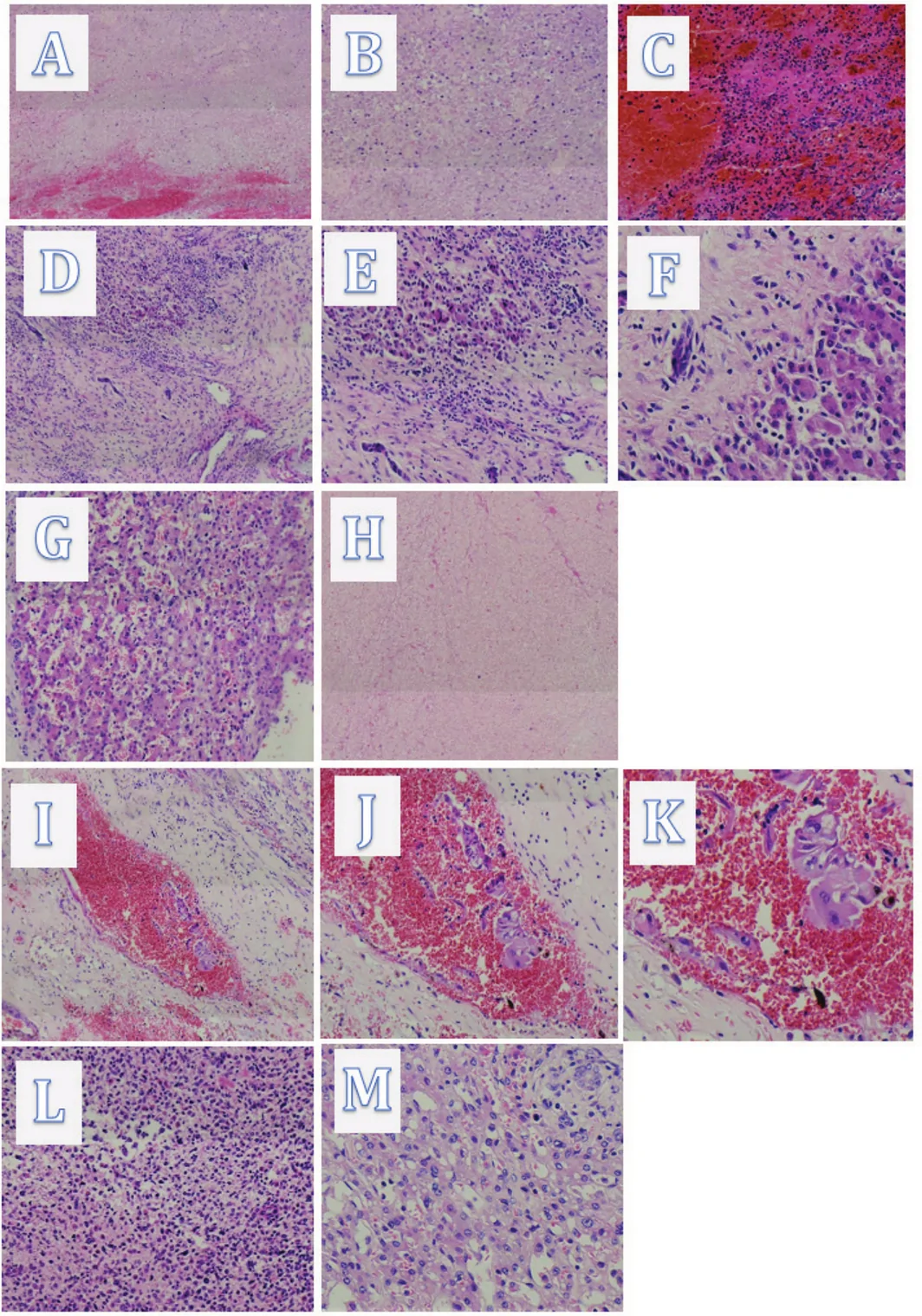
Histopathological findings of the tumor. Photomicrographs demonstrate multiple pathological features of the lesion. Panels A–C show extensive areas of tumor necrosis and hemorrhage at ×10 and ×20 high‐power field (HPF) magnification. Panels D–F illustrate histopathological features consistent with mixed epithelial–mesenchymal teratoid hepatoblastoma, observed at ×10, ×20, and ×40 HPF magnifications, respectively. Panel G demonstrates sinusoidal structures with a trabecular arrangement of tumor cells (×20 HPF), while H shows an area of stromal hyalinization (×20 HPF). Panels I–K depict tumor vascular invasion at ×10, ×20, and ×40 HPF magnifications, respectively. Panel L shows mixed inflammatory cell infiltration, and M demonstrates extravasation of red blood cells within the tumor tissue (both at ×20 HPF).

**FIGURE 4:**
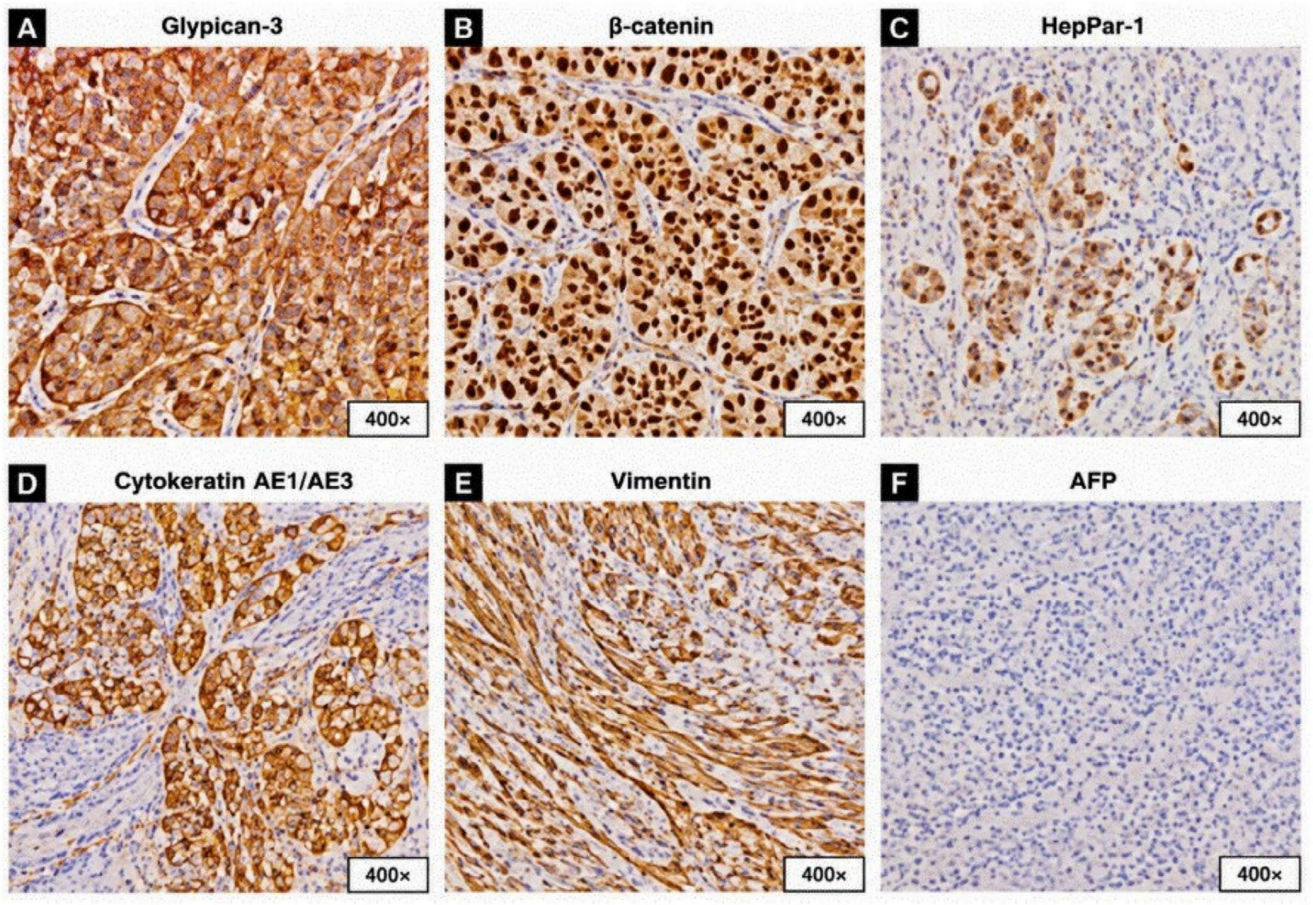
Immunohistochemical findings of the tumor. Photomicrographs demonstrate the immunophenotypic profile of the tumor, supporting the diagnosis of mixed epithelial–mesenchymal teratoid hepatoblastoma. Panel A shows diffuse strong membranous and cytoplasmic positivity for Glypican‐3 (×400). Panel B demonstrates diffuse nuclear and cytoplasmic positivity for β‐catenin (×400). Panel C shows focal cytoplasmic positivity for HepPar‐1 (×400). Panel D highlights the epithelial tumor component with cytokeratin AE1/AE3 immunostaining (×400). Panel E demonstrates strong cytoplasmic positivity for vimentin, highlighting the mesenchymal spindle‐cell component (×400). Panel F shows negative AFP immunostaining in the tumor cells (×400), consistent with the patient's normal serum alpha‐fetoprotein level. The overall immunohistochemical profile supports the diagnosis of mixed epithelial–mesenchymal teratoid hepatoblastoma.

The lesion was considered technically resectable after multidisciplinary review because the future liver remnant was considered adequate and there was no radiological evidence of major vascular encasement or distant metastatic disease.

## 3 Differential Diagnosis, Investigations, and Treatment

The main differential diagnoses considered were hepatoblastoma, hydatid liver cyst, hepatic abscess, other cystic liver tumors, and hepatocellular carcinoma. Initial abdominal ultrasound demonstrated hepatomegaly with a large heterogeneous complex cystic lesion in the right liver lobe. Triphasic contrast‐enhanced CT further characterized the lesion as a large heterogeneous hepatic mass involving the right liver segments, with enhancing septations and a peripheral solid component (Figure 1). There was no evidence of biliary dilatation, portal vein thrombosis, ascites, cirrhosis, or distant metastatic disease in the visualized chest.

Laboratory investigations showed anemia with hemoglobin of 8.9 g/dL and hypoalbuminemia of 24 g/L, while the remaining liver function tests were preserved. Hepatitis B, hepatitis C, and HIV tests were negative. Alpha‐fetoprotein was within the normal range at 1.28 ng/mL, making the diagnosis challenging. Hydatid serology was negative, reducing the likelihood of echinococcal liver disease. Following multidisciplinary evaluation, the patient was planned for a right trisectionectomy. However, clinical deterioration during optimization required emergency surgery. Intraoperatively, a large ruptured hemorrhagic cystic mass with extensive necrosis involving almost the entire right liver lobe was found, with local infiltration into the hepatic flexure of the colon and Gerota's fascia (Figure 3). Right trisectionectomy and radical colectomy were performed en bloc. Histopathological examination confirmed mixed epithelial–mesenchymal teratoid hepatoblastoma with vascular invasion and positive resection margins. Immunohistochemical findings, including diffuse Glypican‐3 and β‐catenin positivity, focal HepPar‐1 expression, cytokeratin AE1/AE3 and vimentin positivity, and negative AFP immunostaining, further supported the diagnosis (Figure 3 and Figure 4).

## 4 Conclusion and Results (Outcome and Follow‐Up)

Postoperatively, the patient was admitted to the intensive care unit for close monitoring and supportive management. However, his clinical course was complicated by disseminated intravascular coagulopathy, and he unfortunately succumbed during the postoperative period. The final diagnosis was ruptured mixed epithelial–mesenchymal teratoid hepatoblastoma in a 10‐year‐old boy with normal alpha‐fetoprotein levels. This case highlights the diagnostic difficulty of hepatoblastoma in older children, particularly when alpha‐fetoprotein is normal, and imaging features overlap with other cystic liver lesions. It emphasizes the need for a high index of suspicion, early multidisciplinary assessment, careful imaging evaluation, and timely surgical intervention in children presenting with complex hepatic masses, even outside the usual age group for hepatoblastoma.

## 5 Discussion

The present case brings together three uncommon and diagnostically challenging features of hepatoblastoma: presentation in an older child, normal alpha‐fetoprotein levels, and tumor rupture. Hepatoblastoma is typically a disease of children younger than 5 years; therefore, its occurrence in a 10‐year‐old boy may lower clinical suspicion and delay definitive diagnosis [3, 4, 5, 6, 7]. The case also demonstrates that a normal alpha‐fetoprotein level does not exclude hepatoblastoma, especially when imaging and histopathological findings support a malignant pediatric liver tumor [7, 9, 10]. This makes the report relevant to clinical practice, particularly in settings where pediatric liver tumors may initially be mistaken for benign cystic, infectious, or inflammatory liver lesions.

The patient presented with chronic right upper quadrant abdominal pain, abdominal distension, early satiety, nausea, vomiting, anemia, and hepatomegaly. These features are consistent with the known clinical spectrum of hepatoblastoma, where abdominal mass or distension is commonly reported, sometimes accompanied by constitutional symptoms and anemia [10, 13, 14]. However, the absence of peritonitis at initial presentation despite later intraoperative evidence of rupture is an important observation. It suggests that tumor rupture may not always present with classic acute abdominal signs, particularly when bleeding or necrosis evolves gradually. This highlights the need for careful clinical monitoring in children with large hepatic masses, even when vital signs are stable and peritoneal signs are absent.

Radiological evaluation was central to narrowing the diagnosis. Ultrasound identified a large complex cystic lesion, while triphasic contrast‐enhanced CT better defined the size, segmental involvement, internal septations, peripheral solid component, and absence of obvious metastatic disease. CT and MRI are essential for defining tumor extent, vascular involvement, local invasion, and surgical planning in suspected pediatric liver tumors [1, 4, 15]. In this case, the predominantly cystic appearance and normal alpha‐fetoprotein complicated the diagnosis and made hydatid cyst, hepatic abscess, and other cystic hepatic tumors important differential diagnoses. Similar diagnostic overlap has been reported where hepatoblastoma mimics infectious or inflammatory liver conditions [16, 17].

Alpha‐fetoprotein is widely used in the diagnosis, risk assessment, and monitoring of hepatoblastoma. In many children, markedly elevated AFP in the presence of a liver mass strongly supports the diagnosis [1, 18]. However, a small proportion of hepatoblastoma cases may have normal or low AFP levels [7, 9, 10]. This case reinforces that reliance on AFP alone may be misleading. When clinical and radiological features remain suspicious, tissue diagnosis through histopathological examination remains essential [1, 19]. The final diagnosis in this patient was only confirmed after resection, with histology showing mixed epithelial–mesenchymal teratoid hepatoblastoma, extensive necrosis and hemorrhage, vascular invasion, and positive margins.

Immunohistochemistry further supported the diagnosis of mixed epithelial–mesenchymal teratoid hepatoblastoma. The tumor demonstrated diffuse positivity for Glypican‐3 and β‐catenin, focal expression of HepPar‐1, cytokeratin AE1/AE3 positivity in the epithelial component, and vimentin expression in the mesenchymal component, while AFP immunostaining was negative. This immunophenotypic profile is consistent with hepatoblastoma and helped confirm the diagnosis in this atypical case, particularly given the patient's normal serum AFP level and the complex cystic radiological appearance [12, 19].

The case also raises important management considerations. Standard hepatoblastoma care depends on accurate staging, assessment of resectability, chemotherapy response, and surgical planning. PRETEXT staging has become the cornerstone of treatment planning in pediatric hepatoblastoma because it objectively defines tumor extent, vascular involvement, and surgical resectability [4, 8, 10, 20, 21, 22]. In our patient, PRETEXT staging suggested locally advanced disease, while emergency presentation with spontaneous rupture prevented the planned neoadjuvant chemotherapy that is recommended in most PRETEXT III/IV tumors. Consequently, immediate surgery became the only life‐saving option despite the increased operative risk.

In many cases, neoadjuvant cisplatin‐based chemotherapy improves resectability and outcomes [8, 23, 24]. However, this patient deteriorated during optimization and required emergency surgery due to tumor rupture. The need for urgent intervention limited the opportunity for planned neoadjuvant therapy and illustrates the complexity of managing ruptured hepatoblastoma in resource‐limited and emergency surgical contexts. The fatal postoperative course was likely influenced by the advanced local disease, tumor rupture, extensive necrosis and hemorrhage, vascular invasion, positive margins, and postoperative disseminated intravascular coagulopathy. These findings suggest aggressive tumor biology and severe systemic physiological stress. Although liver transplantation may be considered for selected unresectable or advanced hepatoblastoma cases [8, 21, 25], emergency presentation with rupture and local infiltration can limit treatment options.

The present case contributes to clinical awareness by showing that hepatoblastoma should remain in the differential diagnosis of complex pediatric liver masses even beyond the usual age group and even when AFP is normal. Early multidisciplinary evaluation, careful imaging interpretation, timely histopathological and immunohistochemical confirmation, and preparedness for emergency surgical complications are essential to improve outcomes in atypical presentations.

## Author Contributions

**Christine Hyasint Paul:** conceptualization, data curation, writing – original draft, writing – review and editing, methodology. **Andrew Swallow:** data curation, conceptualization, writing – original draft, writing – review and editing, methodology. **Vulstan James Shedura:** conceptualization, methodology, data curation, writing – original draft, writing – review and editing. **Albert Lazaro:** writing – original draft, writing – review and editing, methodology, investigation. **Daniel Kitua:** writing – original draft, writing – review and editing, methodology, investigation. **Ayubu Philipo:** writing – original draft, writing – review and editing, methodology, investigation. **Daudi Katwana:** writing – original draft, writing – review and editing, methodology, investigation. **Andrew K. Jackson:** writing – original draft, writing – review and editing, methodology, investigation. **Georvin Haule:** writing – original draft, writing – review and editing, methodology, investigation. **Cameron Gaskill:** methodology, writing – review and editing, writing – original draft, investigation. **Kazungu Boniface Kahindi:** writing – original draft, writing – review and editing, methodology, investigation. **Musa balowa:** writing – original draft, writing – review and editing, supervision, methodology. **Godbless Massawe:** writing – original draft, writing – review and editing, methodology, supervision. **Ally Mwanga:** supervision, writing – review and editing, writing – original draft, methodology.

## Funding

The authors have nothing to report.

## Ethics Statement

The study was approved by the Ethics Committee of the Muhimbili University of Health and Allied Sciences, Dar es Salaam, Tanzania.

## Consent

Written informed consent was obtained from the patient's parent for publication of this case report and any accompanying records. A copy of the written consent is available for review by the editor of this journal.

## Conflicts of Interest

The authors declare no conflicts of interest.

## Data Availability

Data sharing not applicable to this article as no datasets were generated or analyzed during the current study.
